# Quantifying the state-dependent causal effect of Barents–Kara Sea ice loss on the stratospheric polar vortex in a large ensemble simulation

**DOI:** 10.1007/s00382-025-07802-9

**Published:** 2025-08-06

**Authors:** Xiaocen Shen, Marlene Kretschmer, Theodore G. Shepherd

**Affiliations:** 1 Department of Meteorology, University of Reading, Reading, UK; 2 Institute for Meteorology, University of Leipzig, Leipzig, Germany

**Keywords:** Sea ice loss, Stratospheric polar vortex, State-dependence, ENSO, QBO

## Abstract

The Barents–Kara Sea ice concentration (BKS) has undergone dramatic declines in recent decades, consistent with the overall reduction in sea ice across the Arctic region. There has been a long-standing scientific question whether this BKS loss significantly influences winter temperature extremes over mid-to-high latitudes. While there is ongoing debate on this point, it is generally acknowledged that BKS loss affects the stratospheric polar vortex (SPV) through the enhancement of upward propagating waves, which itself can subsequently influence surface weather and climate conditions. However, due to the large internal variability within the climate system and the limited observational data, the strength of the BKS-SPV linkage and its dependence on different background states remain unclear. In this work, we investigate the causal effect of BKS change on SPV using a climate model with large ensemble simulations. Consistent with previous literature, the results indicate that BKS loss significantly weakens the SPV, with the magnitude of the response varying with El Niño‐Southern Oscillation (ENSO) and Quasi-Biennial Oscillation (QBO) phases, indicating a state-dependent causal effect. In particular, El Niño is found to suppress the causal effect of BKS change on the SPV, whereas La Niña and neutral ENSO strengthen it, which is consistent with what is found from observations. In contrast, the effect of QBO alone is relatively weak but becomes more pronounced when combined with ENSO. Dynamical analyses reveal that both tropospheric wave forcing and modulation of stratospheric wave propagation contribute to the state-dependent causal effects. By leveraging large ensemble simulations and combining statistical and physical analyses, this study provides an additional perspective on understanding the factors influencing the SPV response to BKS loss, which could ultimately impact surface climate.

## Introduction

In recent decades, Arctic sea ice concentration and extent have experienced dramatic declines (e.g., Serreze et al. 2007; Stroeve and Notz 2018), especially over the Barents–Kara Seas. It has been a hot topic as to whether the decline in Barents–Kara Sea ice concentration (BKS) has substantial impacts on weather and climate in the mid-latitudes (e.g., McKenna et al. 2018; Xu et al. 2024). Aside from the debate, the stratospheric pathway is recognized as a key pathway capable of establishing a linkage between BKS and mid-latitudes (e.g., Kim et al. 2014; Kretschmer et al. 2016; Screen 2017a; Smith et al. 2019; Zhang et al. 2018a). This is because the response of tropospheric circulation to BKS loss enhances the upward propagation of planetary waves into the stratosphere, thereby weakening the stratospheric polar vortex (SPV, Nakamura et al. 2016; Xu et al. 2021; Zhang et al. 2018b). The changes in the SPV, in turn, can descend into the troposphere, leading to large-scale circulation changes and modifying the surface weather conditions (Kidston et al. 2015; Peings and Magnusdottir 2014; Sun et al. 2015). The response of the SPV to BKS loss is thus of great importance for studying how BKS loss influences mid-latitudes.

**Fig. 1:**
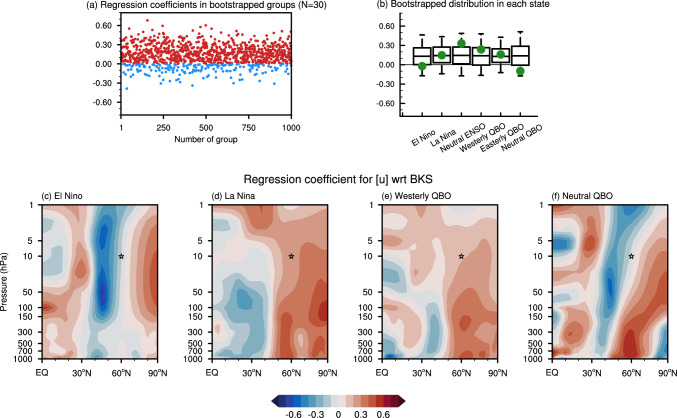
Response of winter mean SPV anomalies to autumn mean BKS anomalies in reanalysis (ERA5) from 1950 to 2023. **a** Regression coefficient for standardized zonal mean zonal wind ([u]) anomalies at 10 hPa, 60°N averaged from December to February with respect to the standardized BKS anomalies ([65°–85° N, 10°–100° E]) averaged from September to November. Each dot represents the result from a bootstrapped subgroup with a sample size of 30 years. Red and blue dots indicate positive and negative values, respectively. **b** Regression coefficients for standardized SPV anomalies with respect to standardized BKS anomalies under different background states (green dots). The box-and-whisker plots show the distribution of the regression coefficients obtained from the bootstrapped data, drawn from the full distribution, but with the same sample size as the samples in the different background states, repeated 1000 times. The 5th, 25th, 50th (median), 75th, and 95th percentiles are shown. **c**–**f** Regression coefficients for standardized [u] anomalies with respect to standardized BKS anomalies under El Niño, La Niña, westerly Quasi-Biennial Oscillation (QBO), and neutral QBO states. The star denotes the location of [u] used as the index of SPV intensity. See the detailed method and definition of ENSO and QBO states in the Data and Methods section

While this stratospheric pathway is dynamically robust, the quantitative response of the SPV to BKS loss shows a large variation (e.g., Seviour 2017; Kretschmer et al. 2020). This variability arises in part from the complexity of the climate system, which is characterized by significant internal variability, and is particularly evident in observations due to the limited sample size (Simon et al.^ 2022^; Warner et al.^ 2020^). For instance, Fig. 1a illustrates the regression coefficients for the standardized winter (DJF) mean SPV anomalies with respect to the standardized autumn (SON) mean BKS anomalies across 1000 bootstrapped subgroups of observational data, each with a sample size of 30 years. The large spread in the regression coefficients highlights how internal variability alone can substantially contribute to the reported intermittent relationship between BKS and SPV (e.g., Siew et al. 2020).

Among the internal variabilities within the climate system, El Niño‐Southern Oscillation (ENSO) and Quasi-Biennial Oscillation (QBO) are the most prominent ones on the interannual timescale. ENSO is characterized by periodic fluctuations between unusually warm and cold conditions in the tropical Pacific (Bjerknes 1969), whereas QBO represents a quasi-periodic transition between easterly and westerly zonal winds in the tropical stratosphere (Baldwin et al. 2001). As both ENSO and QBO can strongly influence the SPV state (Domeisen et al. 2019; Holton and Tan 1980), they have been suggested to significantly modulate the BKS-SPV linkage (Labe et al. 2019; Ma et al. 2022; Xu et al. 2024). To illustrate the varying BKS-SPV linkage, Fig. 1c–f highlight selected states where the relationship appears more pronounced or differs notably from other conditions. Although these plots suggest that observational data reveal clear differences in the BKS-SPV linkage under different ENSO and QBO states, the robustness of these differences is limited by the small sample size in the observations (Fig. 1b). Moreover, as the combined influence of ENSO and QBO might have nonlinear additive effects (Ma et al. 2023; Walsh et al. 2022), the information that the observational record can provide is even further constrained. Hence, there is an emerging need to systematically study the modulation of ENSO and QBO on the BKS-SPV linkage using climate models with large ensembles. Although climate models will invariably contain biases, the results can be used to develop scientific hypotheses which can subsequently be tested against observations, rather than trying to derive the results directly from the observations.

In this work, using the sixth version of the Model for Interdisciplinary Research on Climate (MIROC6) with 50 ensemble members, we aim to quantify the causal effect of BKS loss on the SPV strength under different ENSO and QBO states. Section 2 describes the dataset and the causal framework established in this study. Section 3 analyses how ENSO and QBO modulate the relationship between autumn mean BKS and winter mean SPV. Section 4 presents the causal effects of monthly BKS changes on winter mean SPV under different ENSO and QBO states, followed by the mechanisms responsible for the state-dependent causal effect in Sect. 5. Section 6 presents the combined modulation of ENSO and QBO on the causal effect and the related dynamical processes. A summary and discussion are presented in Sect. 7.

## Data and methods

### Data

Here, we use the historical simulation from the MIROC6 large‐ensemble dataset, which includes 50 members covering the period from 1850 to 2014, making it one of the largest ensembles so far (Shiogama et al. 2023). Monthly data is used and the anomaly for all the variables is computed by extracting the ensemble mean for each year, by which the possible influence of long‐term trends can be excluded. Therefore, this study primarily focuses on interannual variability rather than long-term climate trends. MIROC6 reproduces the QBO reasonably well, and although the QBO-SPV relationship is weaker than observed, it outperforms many current climate models in this aspect (Rao et al. 2020; Richter et al. 2020). In addition, this model generally well simulates the sea ice concentration, despite the slight underestimation in September (Tatebe et al. 2019). However, it is noteworthy that MIROC6 produces an opposite-signed relationship between ENSO and SPV to that in the observations (Manzini et al. 2024; Shen et al. 2024). In the observations, El Niño is typically associated with a weakened SPV, whereas La Niña tends to strengthen the SPV (Domeisen et al. 2019). In MIROC6, however, El Niño leads to a strengthened SPV. Shen et al. (2024) systematically analysed this apparent discrepancy and suggested that this is not necessarily a model bias. The model-observation discrepancy arises from a pathway from ENSO to horizontal wave propagation within the stratosphere, which then dominates the SPV response. Although this pathway is not apparent in observations, the observations also do not provide evidence against its existence. Therefore, this pathway is physically plausible and its absence in observations may result from the limited sample size of the observations. In other words, as discussed in more detail in Shen et al. (2024), it is reasonable to use MIROC6 to study ENSO-SPV coupling. As our main purpose here focuses on the modulation by ENSO of the SPV response to BKS change, rather than the direct linkage between ENSO and SPV, alignment of the ENSO-SPV relationship with observations is not required in this work.

For the observations, monthly mean reanalysis data is obtained from the fifth generation of the European Centre for Medium-Range Weather Forecasts (ERA5) dataset (Hersbach et al. 2020), which extends from 1000 to 1 hPa with 37 layers and is used at a 2.5° × 2.5° horizontal resolution. The monthly averaged sea surface temperature (SST) and sea ice concentration is adopted from the Hadley Centre Sea Ice and Sea Surface Temperature (HadISST) dataset (Rayner et al. 2003). The period being considered spans from 1950 to 2023, with the long‐term trend removed by subtracting the linear trend. The ENSO phase is defined based on the Niño3.4 index, calculated as SST averaged over [5° N–5° S, 120°–170° W] (Trenberth 1997). In the observations, boreal winters in which the absolute value of the Niño3.4 index exceeds 0.5 standard deviations are classified as El Niño or La Niña years accordingly, while the remaining years are classified as neutral ENSO years. Similarly, winters in which the absolute value of equatorial wind ([10°S-10°N]) at 50 hPa exceeds 5 m s^−1^ are classified as westerly or easterly QBO years, with the remaining years classified as neutral QBO years (Fig. 1).

### Statistical framework of the causal linkage and its state dependencies

**Fig. 2:**
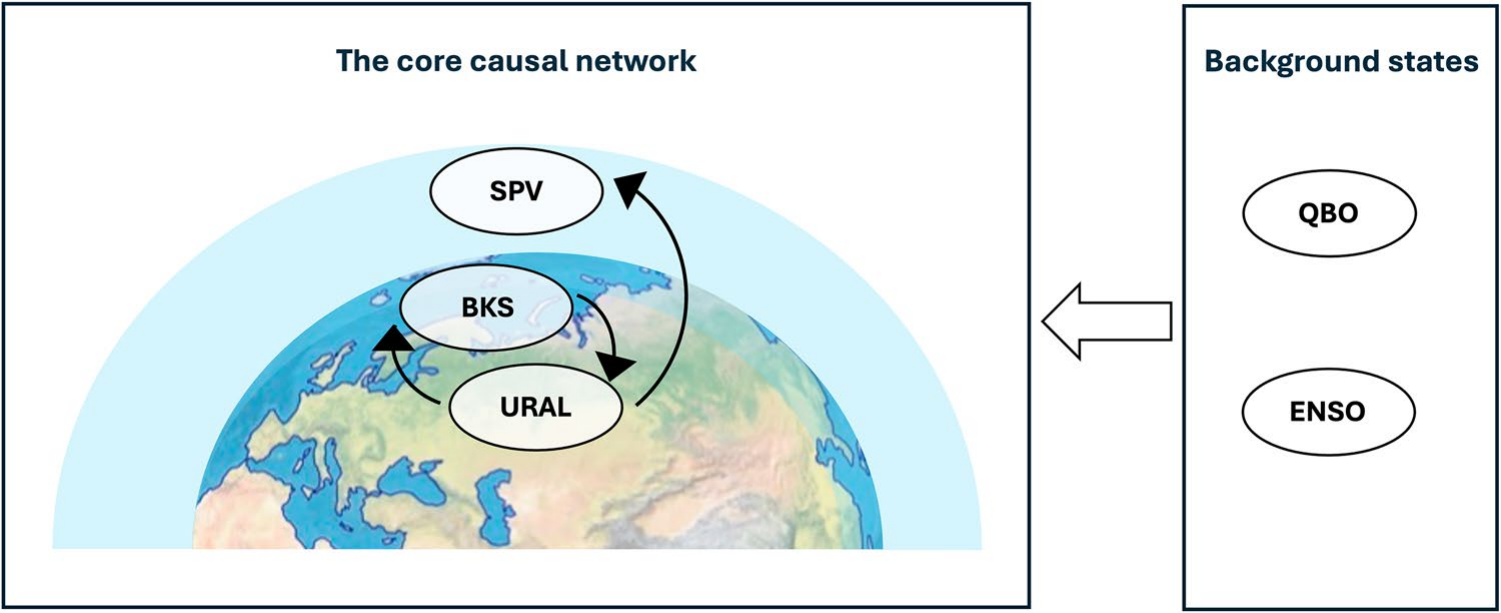
Framework of the causal pathway linking BKS and SPV. The framework includes two steps: quantifying the link based on the core causal network, and additionally computing the link strength given different QBO and ENSO background states. The core causal framework, shown on the left, involves BKS, Ural circulation (URAL), and the SPV, with black arrows indicating the causal relationships among these elements. The background states, listed on the right, include QBO and ENSO, which are hypothesised to modulate the core causal network link strengths

While the correlation coefficient is a useful measurement of linear association, it does not indicate the direction of causality. Conversely, the simple linear regression coefficient, though directional by construction, may fail to estimate the strength of the involved causal pathways due to the presence of confounders. Causal inference provides a more robust framework to quantify teleconnection pathway strengths, as it considers multiple pathways and evaluates their relative strengths (e.g. Kretschmer et al. 2021). To quantify the causal effect of BKS change on SPV and its modulation by ENSO and QBO, this study establishes a two-step causal framework (Fig. 2). The first step is the analysis of what we call the core network, representing the assumed causal linkage between BKS and SPV. Changes in BKS alter heat fluxes over the Barents–Kara Seas, impacting the circulation over the Ural Mountains (URAL), which subsequently influences the SPV through changes in planetary wave propagation (e.g., Kim et al. 2014; Kug et al. 2015; Nakamura et al. 2016). However, URAL circulation can influence both BKS and SPV (e.g., Blackport et al. 2019; Luo et al. 2017), which can generate spurious correlations, i.e. URAL represents a confounding factor (Kretschmer et al. 2016, 2020, 2021). Hence, to quantify the causal effect of BKS change on the SPV, we control for the common driver (URAL_t−1_) by including it in the following multivariate linear regression (MLR) model:

1 $$ SPV_{DJF} = \alpha \cdot BKS_{t} + \beta \cdot URAL_{t - 1} + residual $$

Here $\alpha$ represents the causal effect of BKS changes on the SPV, and $\beta$ quantifies the influence of URAL circulation on the SPV that is not mediated by BKS (see also Kretschmer et al. 2016, 2020, 2021). In this study, we consider BKS changes from individual months (September to November, $t$ in Eq. 1) and their influence on December to February (DJF) mean SPV intensity. The URAL circulation one month prior to the BKS change ($t - 1$ in Eq. 1) is included in the MLR model to control for its confounding effect on BKS. Here, the SPV intensity is represented by the zonal mean zonal wind ([u]) at 10 hPa, 60°N (e.g., Charlton and Polvani 2007), BKS is represented by the sea-ice area percentage (siconc) averaged over [65°–85° N, 10°–100° E] (Screen 2017b), and the URAL circulation is represented by sea level pressure averaged over [45°–70°N, 40°–85° E] (Kretschmer et al. 2016, 2020). Unless specified otherwise, all indices are first standardized before subgrouping (i.e., to zero mean and unit variance), thus allowing for comparison across different states.

The modulation of the background states is then considered in the second step of our framework, where we repeat the calculation of the causal linkage but in different subgroups based on the states of ENSO, QBO, and their combination. The ENSO states are quantified using the Niño3.4 index. Given the lagged response of the SPV to ENSO (Domeisen et al. 2019; Garfinkel and Hartmann 2008), the November-January Niño3.4 index is used to represent winter ENSO conditions. ENSO states are categorized based on a ± 0.5 standard deviation ($\sigma$) threshold. For QBO, we use Empirical Orthogonal Function (EOF) analysis to capture the vertical profile of the stratospheric equatorial [u] (Baldwin and Dunkerton 1998; Wallace et al. 1993). We apply EOF analysis to the 10° S–10° N averaged [u] with the range from 100 to 10 hPa to capture the vertical structure of the QBO (Coy et al. 2022). The first two leading EOFs obtained in the MIROC6 closely resemble observations, albeit with weaker amplitudes of wind velocity (not shown). This is consistent with previous studies showing that the QBO in MIROC6 is weaker than observed (e.g., Rao et al. 2020; Richter et al. 2020). Despite this model bias, MIROC6 outperforms most other models and is therefore suitable for further analysis.

**Fig. 3:**
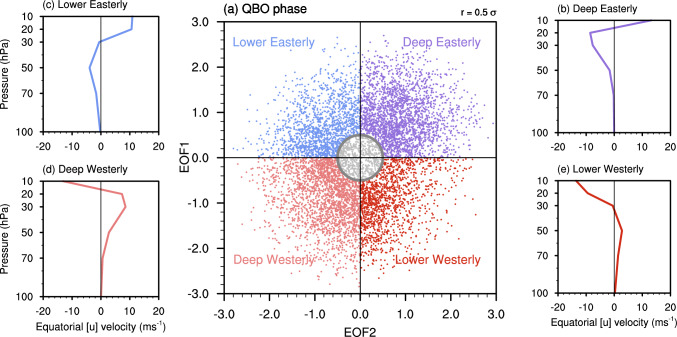
Distribution and wind profiles of different QBO phases. **a** Distribution of the winter mean QBO phase. The different colours represent different QBO phases. The grey dots indicate winters where the amplitude is smaller than 0.5. The circle indicates the range where the amplitude equals 0.5. **b** The zonal mean zonal wind in the equatorial stratosphere in the deep easterly QBO phase, as a function of pressure. **c**–**e** Same as **b** but for the lower easterly QBO, deep westerly QBO, and lower westerly QBO phase, respectively

As the vertical structure of the zonal wind is essential for the QBO to influence the SPV (Andrews et al. 2019) and we have a large sample size available to investigate the modulation from different QBO wind structures, four QBO phases are defined in this work based on the distribution of the associated principal components (PCs, Fig. 3a). The deep easterly and lower easterly phases represent the easterly descending phases of the equatorial wind in the middle and lower stratosphere, with the easterly wind maxima around 20 hPa (~ 8 m s^−1^) and 50 hPa (~ 4 m s^−1^), respectively (Fig. 3b, c). The deep westerly and lower westerly phases, on the contrary, show the westerly descending phases with westerly maxima around 30 hPa (~ 9 m s⁻^1^) and 50 hPa (~ 3 m s⁻^1^), respectively (Fig. 3d, e). The QBO states are classified based on the DJF mean value, with the amplitude defined as the square root of the sum of the squared PC1 and PC2 values. Winters with an amplitude exceeding a 0.5 threshold are classified as specific QBO phases, while the remaining states are classified as neutral QBO states. Note that, due to the oscillating nature of the QBO, only a limited number of years are classified as neutral QBO states (grey dots in Fig. 3a). Therefore, these neutral QBO states are not considered in the analysis. The above threshold values are selected to maximize sample size, with the number of each group labelled in the related figures. We also tested the sensitivity of the QBO structure and phase distribution to the choice of vertical range by using 70–10 hPa instead of 100–10 hPa, and found no perceptible difference (not shown).

## Influence of ENSO and QBO states on SPV and BKS-SPV causal linkage

### Influence of the background states on the SPV

**Fig. 4:**
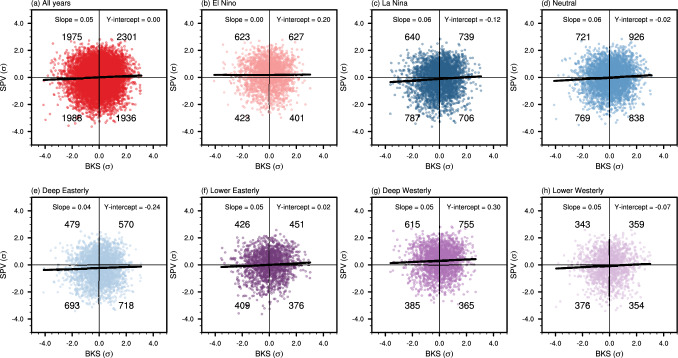
Scatter plots of BKS and SPV under different ENSO and QBO states. **a** BKS (x-axis) and SPV (y-axis) for all years. **b**–**d** Same as **a**, but for different ENSO states. **e**–**h** Same as **a**, but for different QBO states. The number in each quadrant indicates the corresponding count of data points. The bold line represents the linear regression fit

**Fig. 5:**
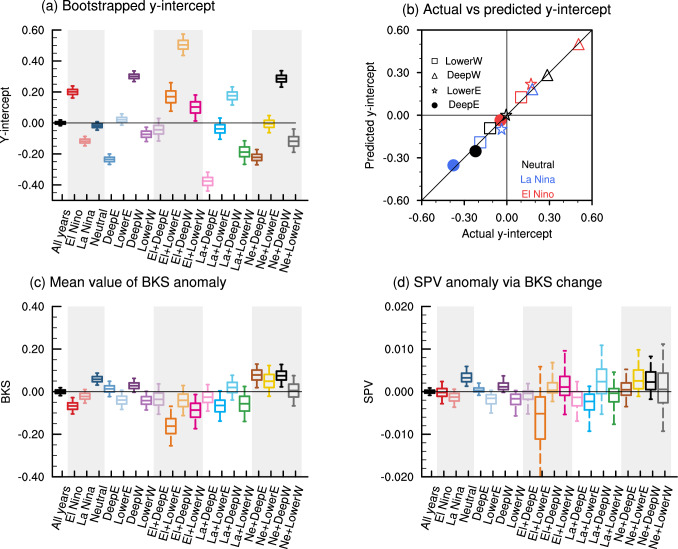
Impact of background states on December-February mean SPV, September–November mean BKS, and BKS-contributed changes in SPV. **a** Distribution of the y-intercept from the BKS-SPV scatter plot shown in Fig. 4, representing the mean SPV anomaly under different background states, excluding the contribution from BKS. The distribution is generated by bootstrapping samples within each background states with replacement, repeated by 1000 times. Colours represent different background states, with grey shading added for visual clarity between categories. **b** Actual (x-axis) and predicted (y-axis) y-intercepts for ENSO-QBO combined states. The actual values follow the pattern shown in Fig. 4, while the predicted y-intercepts are calculated as the linear sum of the y-intercepts from the corresponding individual QBO and ENSO states. Colours denote ENSO states, and marker shapes indicate QBO phases. The black solid line denotes the line where predicted values equal actual values (i.e., y = x). **c** and **d** Same as **a** but showing the distributions of the BKS anomaly and the SPV anomaly induced by BKS changes, respectively. Note the differing y-axis scales for visualization purposes and note especially the difference in scale between **a** and **d**

To examine the causal linkage between BKS and SPV under different background states, we first present the scatter plot of standardized autumn (SON) mean BKS and standardized winter (DJF) mean SPV in Fig. 4. Here, the influence of preceding URAL circulation (August to October mean) on the SPV has been removed, following the causal framework outlined in Fig. 2. For all years (i.e., the unconditional state), the slope of the linear fit is positive, indicating that a decrease in BKS is associated with a weakening of the SPV (Fig. 4a). In addition, the y-intercept is close to zero. However, when the data are stratified by ENSO states, the y-intercept changes to positive in El Niño years (0.20), to negative in La Niña years (− 0.12), and to slight negative in neutral ENSO years (− 0.02). Note that all data were first standardized before classification into subgroups, thus allowing for comparison across different states. These changes indicate that, under different ENSO states, the SPV intensity independent of BKS change shows clear differences. Specifically, the SPV tends to be stronger in El Niño years and weaker in La Niña years. While this differs from the conventionally recognized negative ENSO-SPV linkage, it is not necessarily a model bias as discussed in Sect. 2.1. To assess the magnitude of these y-intercept changes, Fig. 5a shows the distribution of the y-intercepts after bootstrapping with replacement within each subgroup. The results confirm the varying SPV responses across different states, thus highlight a strong state-dependence in SPV intensity.

Similarly, across different QBO phases, there are clear changes in the y-intercept, with negative values during deep easterly phase and positive values during deep westerly phase (Figs. 4e, g and 5a), consistent with the Holton-Tan effect (Holton and Tan 1980). The emergence of this linkage predominantly under deep QBO structures further suggests a stronger influence of the deep QBO on SPV variability (e.g., Andrews et al. 2019; Wan et al. 2025). When ENSO and QBO phases are combined, the changes in y-intercept are even more pronounced (Fig. 5a). A closer inspection suggests that the combined influence of ENSO and QBO on the SPV appears highly linear. To evaluate this linearity, Fig. 5b compares the actual y-intercepts (x-axis) with the predicted counterparts (y-axis), calculated by summing the y-intercepts from the individual ENSO and QBO states. This close alignment between the predicted and actual values suggests a strong linear interaction between ENSO and QBO in their joint influence on the SPV. This finding is consistent with Wang et al. (2024), who analyzed multiple climate models. However, it differs from Walsh et al. (2022), which reported a nonlinear joint influence on the SPV using a single model.

In line with changes in the y-intercept, the distributions of scatter points also vary across different background states. For all years, the points are evenly distributed across the four quadrants, as expected. However, in El Niño years, more points appear in the upper quadrants, while in La Niña years, they concentrate in the lower quadrants (Fig. 4a–c). This vertical shift in distribution is statistically significant at the 95% confidence level, based on a binomial distribution. Similarly, the vertical shift is also significant across different QBO phases. In contrast, the horizontal distribution remains relatively consistent across states, indicating that the mean BKS anomaly does not vary substantially under different ENSO and QBO states. This is further supported by Fig. 5c, which shows that the distribution of mean BKS anomalies has a much smaller magnitude than that of the y-intercept. Nevertheless, clear variations still exist. For instance, while the median value for all years is zero, it shifts to negative values during El Niño and La Niña years, and becomes positive in neutral ENSO years. It has been suggested that the impact of ENSO on BKS is modulated by the URAL circulation (e.g., Luo et al. 2023). However, after regressing out the URAL circulation, the distribution of BKS does not change significantly (not shown). A question that naturally arises is the extent to which the state-dependent BKS mean anomaly contributes to the state-dependent SPV mean anomaly. This can be partly addressed by examining the product of the mean BKS anomaly and the regression coefficient of SPV with respect to BKS, which is shown in Fig. 5d. The result indicates that this contribution can hardly explain the sensitivity of SPV, confirming that the strong state-dependence of SPV to ENSO and QBO is independent of BKS. This is to be expected, as the well-established pathways for ENSO and QBO to influence the SPV are not directly linked to BKS.

### Influence of the background states on the BKS-SPV causal linkage

**Fig. 6:**
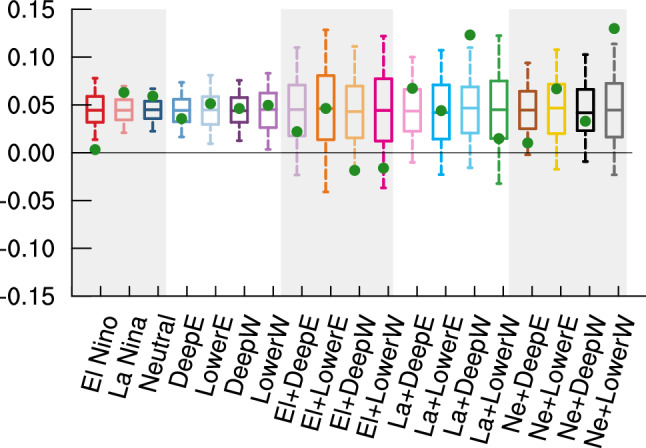
Distribution of regression coefficients for the BKS-SPV linear relationship under different background states. Green dots indicate the actual values, whereas the box-and-whisker plots show the distribution of regression coefficients obtained via bootstrapping, using the same sample size as that of the respective background states. Note that this bootstrap approach differs from that used in Fig. 5, as here we resample the unconditional dataset to evaluate whether the actual values arise from sampling uncertainty. Colours indicate different background states. The grey shading is added to visually separate the categories

Another apparent feature from the scatter plots is the variation in the slope of the linear regression line (i.e., the regression coefficient), which reflects the linear causal linkage between BKS and SPV (Fig. 4a–h). The slope is approximately 0.05 when considering all years, but it drops to near zero in El Niño years, and increases to around 0.06 in both La Niña and neutral ENSO years (Fig. 4a–d). This difference indicates that the BKS-SPV causal linkage is mainly active in La Niña and neutral ENSO years but diminishes in El Niño years. For different QBO states, the slope remains generally consistent, with a slightly weaker value in the deep easterly QBO phase (Fig. 4e–h). The statistical significance of these slope changes can be evaluated by comparing the observed values with the bootstrapped distribution generated from the full data, where random samples are selected without replacement using the same sample sizes as those in each background state. As shown in Fig. 6, the slope in El Niño years falls below the 5th percentile of the bootstrapped distribution, indicating a significant difference to the all-years condition. In contrast, the slopes for La Niña and neutral ENSO states exceed the 75th percentile. While the slope changes across QBO phases are less pronounced, their joint influence with ENSO can produce robust effects. For instance, in the deep westerly QBO phase combined with La Niña and in the lower westerly QBO phase combined with neutral ENSO, the causal effects are significantly stronger than the all-years condition.

The above analysis also justifies our two-step causal framework and the hybrid approach that combines linear regression with categorical background states. The linear assumption serves as a first-order and practical method for investigating the linkage between two variables, including BKS and SPV (e.g., Kretschmer et al. 2020). It also provides valuable insights into the state-dependence of both the BKS-SPV relationship (reflected in the slope) and of the mean SPV anomaly (reflected in the y-intercept). In terms of the background state classification, ENSO shows a clear asymmetric influence on both the y-intercept (Figs. 4 and 5a) and the BKS-SPV causal linkage (Fig. 6). This highlights the need to consider the influence of different ENSO states, rather than including the ENSO index in the linear regression. Similarly, categorizing QBO phases is also necessary, especially since our aim is to explore the influence of different QBO structures. Thus, applying linear regression within categorical states is a reasonable and effective strategy for this study. A similar hybrid approach has also been used in previous studies, such as Di Capua et al. (2019) and Saggioro et al. (2020).

## Causal effect of monthly BKS on winter mean SPV under different ENSO and QBO states

**Fig. 7:**
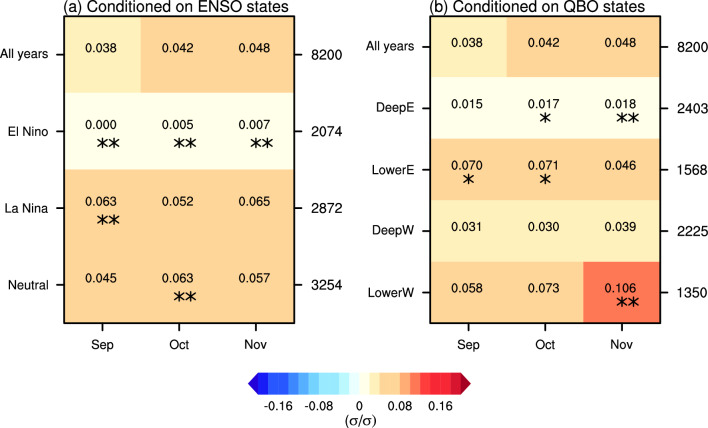
Standardized causal effect of BKS on SPV under different ENSO and QBO states. **a** Causal effects in different ENSO states. The x-axis represents the month of BKS change, while the y-axis indicates different background states. **b** Same as **a**, but for QBO states. The numbers on the right side of each panel denote the sample size (i.e., the number of years) for each state. One and two stars indicate that the causal effect differs from the result for all years at the 90% and 95% confidence level, respectively. Statistical significance is assessed based on the bootstrapped distribution with the same sample size as each state, repeated 1000 times

Given that ENSO and QBO exhibit subseasonal variations in their influence on the SPV (e.g., Anstey and Shepherd 2014; Manzini et al. 2006), and that sufficient sample size is available, we then further examine the causal effects of monthly BKS anomalies on winter-mean SPV. The causal effect of BKS changes on the SPV, unconditioned by background state (i.e., for all years), is first presented in the top row of Fig. 7a. The results show that BKS changes in September, October, and November all generate positive causal effects on the winter SPV, with values of 0.038, 0.042, and 0.048, respectively. These positive causal effects suggest that BKS loss leads to weakening of the SPV in MIROC6, consistent with the observations and theoretical expectations (Kim et al. 2014). The quantified results mean, for example, that a one standard deviation (σ) decrease in September BKS results in a 0.038 σ weakening of the winter mean SPV. Although the magnitudes of these causal effects are small, their influence is physically significant given the large variability of the SPV and the substantial magnitude of BKS decrease in the past (Cosford et al. 2025). Moreover, they carry important implications for future projections (Kretschmer et al. 2020). For instance, based on MIROC6 projections under the SSP585 scenario, by the end of the twenty-first century, October BKS is expected to decrease by 7.2 σ, and thereby contribute to a − 0.3 σ change in SPV, equivalent to a 2.1 m s^−1^ decrease in wind speed. Such a change can be expected to exert a substantial influence on surface climate and weather conditions (Manzini et al. 2014; Simpson et al. 2018). It is also noteworthy that while the observation-based estimate is larger, these causal effects are generally consistent with results averaged across CMIP5 models (Kretschmer et al. 2020), further supporting the robustness of the causal effect of BKS change on SPV based on the MIROC6 large ensemble.

These causal effects change significantly under different background ENSO states (Fig. 7a). In El Niño years, the causal effects decrease substantially throughout the autumn season, with values dropping to 0.000, 0.005, 0.007 for September, October, and November, respectively. In contrast, during La Niña and neutral ENSO years, the causal effects strongly increase, by up to 0.063 which is roughly a 50% increase. Here the significance is determined using the bootstrap approach similar to that used in Fig. 6. For example, to evaluate whether the causal effect in El Niño years significantly differs from the result for all years, a bootstrap resampling without replacement is conducted using the full dataset of 8200 years, with the sample size matching that of El Niño years (i.e., 2074 years). The causal effect in El Niño years is then compared against the distribution of bootstrapped results to determine if it represents an outlier unlikely to arise from internal variability alone. The overall pattern closely resembles the seasonal mean BKS influence shown in Fig. 6.

Under different QBO phases, the causal effects also show substantial variation (Fig. 7b). In the deep easterly and deep westerly QBO phases, the causal effects decrease compared to the results for all years. In contrast, in the lower easterly and lower westerly QBO phases, the causal effects increase significantly. For example, the value for November BKS under the lower westerly QBO phase rises to 0.106, more than twice the original value (0.048). Interestingly, the sensitivity to QBO phase is not primarily between easterly and westerly phase but between phases which peak in the upper or lower stratosphere. This sensitivity to QBO phase of the causal linkage between monthly BKS and winter mean SPV is in contrast to the results for seasonal mean BKS (Fig. 6), which show a much weaker effect. This indicates that the effect of the QBO on the BKS-SPV linkage, like the effect of the QBO on the SPV itself, has a strong subseasonal dependence.

## Dynamical mechanisms responsible for the state-dependent causal effect

To address why the causal effect of BKS on SPV is state-dependent on ENSO and QBO, in this section we analyse the involved dynamical mechanisms. To isolate the changes induced by BKS change, we use the same MLR approach to regress out the influence of the confounding factor, namely the antecedent changes in the URAL circulation. We focus on the impact of October BKS change, noting that the dynamical processes are not quantitatively different in other months (not shown). The MLR is expressed in the form of:

2 $$ Dynamical \,variable_{DJF} = \gamma \cdot BKS_{Oct} + \delta \cdot URAL_{Sep} + residual $$

Here, $\gamma$ represents the causal linkage between October BKS and the dynamical variables under consideration, which include zonal mean zonal wind ([u]), EP-flux, EP-flux divergence, and geopotential height at 300 hPa. The regression coefficient $\delta$ quantifies the influence of URAL on the dynamical variable not mediated by BKS. Here, the EP-flux is calculated based on monthly means under the quasi-geostrophic approximation, thus representing propagation of the stationary waves (Andrews et al. 1987; Edmon et al. 1980). Note that all dynamical variables, apart from BKS and URAL indices, are non-standardized to allow for further budget analysis. The value of the regression coefficient $\gamma$ has been multiplied by − 1 in the following analysis to better interpret scenarios where BKS decreases.

**Fig. 8:**
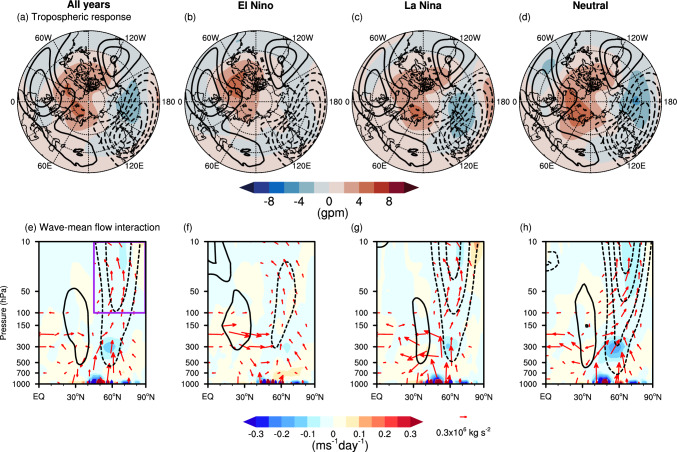
Dynamical picture of the causal linkage between October BKS loss and SPV under different ENSO states. **a–d** Geopotential height anomaly at 300 hPa in response to a 1 σ BKS loss (shading, unit: gpm). Contours represent the climatological stationary waves, calculated as the climatological geopotential height with the zonal mean removed (contour interval is 40 gpm). **e**–**h** Zonal mean zonal wind ([u], contours, contour interval is 0.1 m s^−1^), EP-flux (arrows) and EP-flux divergence (shading, unit: m s^−1^ day^−1^) anomalies in response to a 1 σ BKS loss. The rows, from left to right, show results for all years, El Niño years, La Niña years, and neutral ENSO years, respectively. The vertical component of EP-flux has been multiplied by 100 for visualization purposes. The purple box in **e** denotes the region used for EP-flux budget analysis ([45°–90° N, 100–10 hPa])

Figure 8a illustrates the tropospheric circulation response to October BKS loss without conditioning on background states. There is a clear anticyclonic anomaly centred over the BKS region and a cyclonic anomaly belt located within the 45°–75°N latitude band (shading). The climatological stationary wave pattern (contours) presents a cyclone over the North Pacific and anticyclones over the west coast of North America, the North Atlantic, and the Ural region. Therefore, the anomalous circulation pattern induced by BKS loss constructively interferes with the climatological stationary waves, enhancing upward propagation of the planetary waves into the stratosphere (arrows in Fig. 8e). This upward wave propagation leads to EP-flux convergence in the polar stratosphere, resulting in zonal wind deceleration, and correspondingly, a weakened SPV. This process aligns well with the established dynamical mechanisms by which BKS change influences the SPV (e.g., Kim et al. 2014; Xu et al. 2024).

In El Niño years, although the overall tropospheric circulation response is similar, the cyclonic anomaly over the North Pacific is much weaker (Fig. 8b). This results in a weaker upward wave propagation and, consequently, a weaker SPV anomaly (Fig. 8f). In contrast, during La Niña and neutral ENSO years, the cyclonic anomaly over the North Pacific is stronger than in all years (Fig. 8c, d), and is strongest in the neutral ENSO condition (Fig. 8d). As a result, upward wave propagation is strongest under neutral ENSO conditions and second strongest in La Niña years. Apart from the vertical wave propagation, another remarkable difference is that only in the neutral ENSO states is there poleward wave propagation within the stratosphere, which also contributes to the EP-flux convergence (Fig. 8h). Consequently, the causal effect is greatest in neutral ENSO conditions, followed by La Niña years, and then El Niño years, which exhibit the smallest causal effect (Fig. 7a, second row). For the responses to BKS loss in other months, while the quantified results vary, the underlying dynamical processes remain similar (not shown).

To quantify the contribution of wave propagation to the modulation of the causal effects, we further apply an EP-flux budget analysis, in which the EP-flux is integrated across specific boundaries to assess the relative contributions (e.g., Sigmond and Shepherd 2014). Specifically, this budget is conducted in the polar stratosphere, covering the range of [45°–90° N, 100–10 hPa] (purple box in Fig. 8e, Kushner and Polvani 2004). The integration of the vertical component of EP-flux (Fz) at 100 hPa represents the stratospheric wave forcing, with positive values indicating upward wave propagation into the stratosphere. Similarly, the integration of Fz at 10 hPa reflects wave propagation into the upper stratosphere. In addition, Fz at 300 hPa is integrated to represent tropospheric wave forcing, which is more directly linked to tropospheric circulation anomalies (Yessimbet et al. 2022). At the southern boundary, the integration of the meridional EP-flux (Fy) characterizes horizontal wave propagation within the stratosphere, where positive values denote poleward propagation and negative values indicate equatorward propagation.

**Fig. 9:**
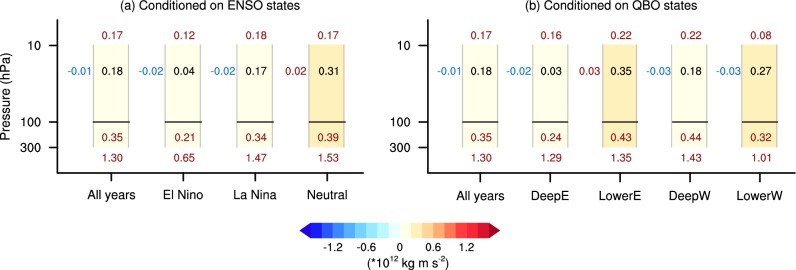
Budget of EP-flux convergence anomalies across different states. **a** Budget for groups conditioned on ENSO states. **b** Budget for groups conditioned on QBO states. The black numbers indicate the EP-flux convergence anomaly integrated over the chosen domain (purple box in Fig. 8e) in response to a 1 σ BKS loss, with the background colour representing its range, as illustrated by the colour bar. The numbers in the vertical direction correspond to the integrated Fz anomalies at the upper boundary (10 hPa), lower stratosphere (100 hPa), and upper troposphere (300 hPa), listed from top to bottom. The values on the left denote horizontal wave propagation (Fy) within the stratosphere. The colour of the numbers indicates the sign of the integrated EP-flux at each boundary, with red representing positive values and blue representing negative values

Figure 9a shows the results of the EP-flux budget across different ENSO states, represented by each column. For easy comparison, the black numbers represent the EP-flux convergence in the polar stratosphere, with the opposite sign to the divergence shown in Fig. 8. The EP-flux convergence is calculated as the sum of the convergence over the selected domain; therefore, it does not quantitatively equal the sum of the contributions from each boundary. The numbers on the left correspond to horizontal wave propagation (Fy), and other numbers indicate the integrated Fz values for waves propagating into the upper stratosphere (10 hPa), the stratospheric wave forcing (100 hPa), and the tropospheric wave forcing (300 hPa). The results reveal that the EP-flux convergence is strongest in the neutral ENSO state, which is responsible for the most pronounced weakening of the SPV (i.e., the strongest causal effect). This strongest EP-flux convergence is on the one hand driven by the strongest stratospheric wave forcing, which originates from the strongest tropospheric wave forcing; on the other hand, it also benefits from poleward wave propagation within the stratosphere, consistent with the results shown in Fig. 8. For El Niño years, the weakest tropospheric wave forcing directly results in the weakest stratospheric wave forcing, leading to the smallest causal effect (Fig. 7a). Notably, while the tropospheric wave forcing in La Niña years is stronger than in all years, the stratospheric wave forcing is slightly higher in all years than in La Niña years. This indicates that, although the variability in tropospheric wave forcing predominantly explains the variability in stratospheric wave forcing, the latter can also vary independently (Christiansen 1999; Scott and Haynes 2000; Yessimbet et al. 2022).

**Fig. 10:**
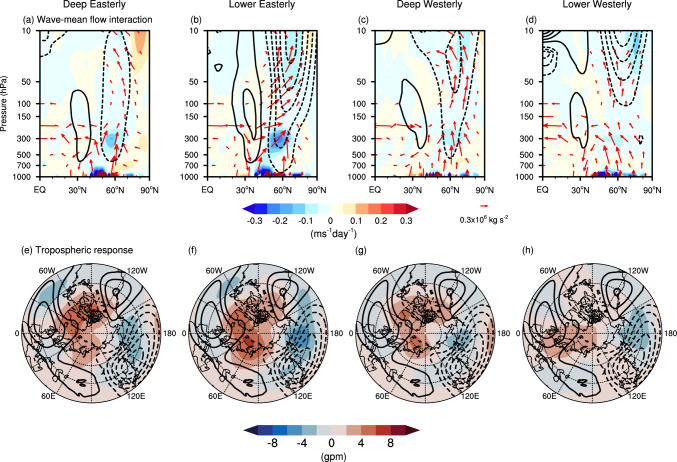
Dynamical picture of the causal linkage from BKS loss in October on SPV in different QBO states. Same as Fig. 8 but for the response to BKS loss conditioned on different QBO phases

We next apply these analyses to different QBO phases. As the QBO is known to influence the SPV primarily by modulating horizontal wave propagation within the stratosphere (e.g., Anstey et al. 2022; Garfinkel et al. 2012; Holton and Tan 1980), we first check the dynamical picture of the wave-mean flow interaction. Figure 10a–d illustrate the EP-flux and [u] anomalies in response to BKS loss across the QBO phases. Substantial differences emerge in horizontal wave propagation within the stratosphere. During the deep westerly and lower westerly QBO phases, when the equatorial [u] is in a westerly descending phase in the middle and lower stratosphere (Fig. 3d, e), the anomalous waves induced by BKS loss tend to propagate equatorward within the stratosphere (Fig. 10c, d). Conversely, during the lower easterly phase (Fig. 3c), these waves propagate poleward (Fig. 10b). This behaviour is broadly consistent with the classical Holton-Tan relationship (Holton and Tan 1980), where the QBO modulates wave propagation once the waves enter the stratosphere. This supports the idea that the QBO modifies the background state, thereby influencing how the BKS-induced wave anomalies propagate. However, during the deep easterly QBO phase, this relationship appears to break down. Despite an easterly wind profile (Fig. 3b), the waves propagate equatorward (Figs. 9b, 10a). This suggests that the vertical structure of the QBO plays an important role in modulating the propagation of waves in response to BKS loss. Moreover, while the distinct horizontal wave propagation can explain the strong EP-flux convergence in lower easterly QBO and weak convergence in deep easterly and deep westerly QBO phases (Fig. 9b), it does not account for why the EP-flux convergence in the lower westerly QBO is stronger than in deep easterly and deep westerly QBO phases.

The resolution of this puzzle is that upward wave propagation varies across QBO phases. For the tropospheric wave forcing, the intensity varies despite the overall consistent tropospheric circulation responses (Fig. 10e–h). There is stronger constructive linear interference with the climatological stationary waves in the lower easterly and deep westerly QBO phases (Fig. 10f, g), resulting in stronger tropospheric wave forcing and, consequently, strong stratospheric wave forcing in these two phases (Fig. 9b). In the lower westerly phase, the combination of strong tropospheric wave forcing and poleward wave propagation together leads to the strong EP-flux convergence and, consequently, the strong causal effect (Fig. 7b). Although the deep westerly QBO phase exhibits similarly strong stratospheric wave forcing, the waves propagate further upward into the upper stratosphere. Combined with the equatorward propagation in the horizontal direction, this leads to weaker wave convergence and a weaker causal effect (Figs. 7b and 9b). In contrast, in the lower westerly QBO phase, despite weaker tropospheric wave forcing, the stratospheric wave forcing is comparable to all years. Moreover, the waves are confined to the middle stratosphere, with the weakest upward propagation into upper stratosphere among different states (Fig. 10d). As a result, the EP-flux convergence and the resulting causal effect is stronger. In the deep easterly QBO phase, weak stratospheric wave forcing directly leads to the weakest wave convergence. However, this is unlikely to originate solely from troposphere wave forcing, which is comparable to all years.

These results suggest that the QBO influences the BKS-SPV causal linkage through complex processes, including modulation of both the tropospheric circulation induced by BKS loss and the propagation of waves once they enter the stratosphere. This is expected, as the QBO can influence tropospheric circulation via various pathways (Anstey et al. 2022; Gray et al. 2018), which in turn can affect vertical wave propagation and ultimately the SPV. As a result, while the lower easterly and lower westerly QBO phases exhibit opposite wind profiles, both lead to a strengthened causal effect. In addition, although deep easterly and lower easterly QBO phases both represent descending phases of QBO easterlies, the dynamical processes and the quantified causal influences differ significantly. This is also the case for the deep westerly and lower westerly phases. The different modulation in each case confirms that the vertical structure of the QBO wind profile plays a critical role in exerting climate impacts. Overall, these findings underscore that the influence of QBO on the climate system is complex and suggest that incorporating the vertical structure of QBO winds could provide further insights.

## Combined modulation of ENSO and QBO states on the causal linkage

**Fig. 11:**
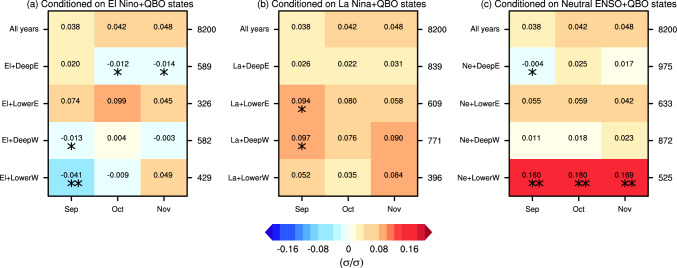
Causal effect of BKS changes on the SPV conditioned on combinations of ENSO and QBO states. **a**–**c** Same as Fig. 7 but for the causal effects conditioned on El Niño combined with QBO phase, La Niña combined with QBO phase, and neutral ENSO combined with QBO phase, respectively

With the large sample sizes provided by the large ensemble simulation, it is feasible to analyse the combined influence of ENSO and QBO on the causal effect of BKS change on the SPV. Figure 11 shows the effect estimated across various ENSO-QBO combinations. Dividing the data into more subgroups reduces the sample size greatly, leading to overall less robust results. Nevertheless, there are still some significant modulations. For example, in the combination of neutral ENSO with lower westerly QBO phase (fifth row in Fig. 11c), the causal effect increases significantly, up to a value of 0.18 for October BKS change—more than four times the original value (0.042). This result aligns with expectations, as both neutral ENSO and lower westerly QBO amplify the causal effect of BKS on the SPV (Fig. 7). However, it is surprising that some combinations yield negative causal effects, such as El Niño with deep easterly, deep westerly and lower westerly QBO phase, and neutral ENSO with deep easterly QBO phase. In addition, although the lower westerly QBO phase strengthens the causal effect and the deep westerly phase weakens it, their combination with La Niña leads to comparable causal effects (Fig. 11b). This further highlights the intricate and nonlinear interactions between ENSO and QBO states in modulating the causal effects of BKS change on the SPV.

**Fig. 12:**
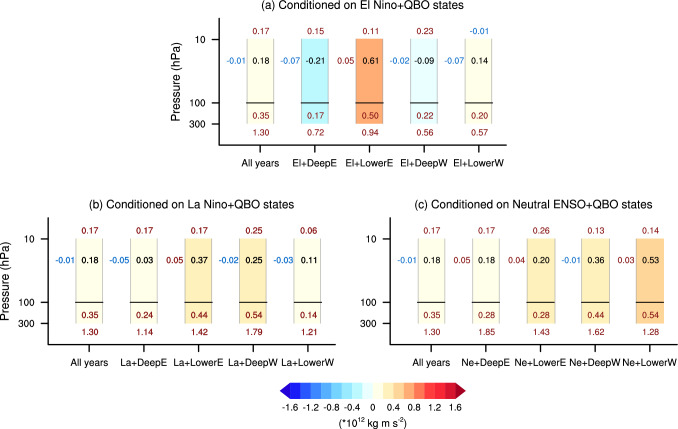
Budget of EP-flux convergence in ENSO and QBO combined state. Same as Fig. 9 but for combined ENSO and QBO background states

The dynamical mechanism underlying these findings is explored in Fig. 12. Overall, the nonlinear interaction between ENSO and QBO on the BKS-SPV causal linkage is evident. For instance, while the tropospheric circulation response to October BKS loss is strongest in the deep westerly QBO phase compared to other QBO phases (Fig. 9b), this relationship shifts when combined with El Niño, where the strongest response occurs in the lower easterly QBO phase (Fig. 12a). Although the tropospheric wave forcing in the lower easterly QBO phase and El Niño combined state remains weaker than all years, the presence of strong stratospheric wave forcing leads to the greatest causal effect among the QBO phases when combined with El Niño (Fig. 11a). For the horizontal wave propagation, while the wave propagation across different QBO states combined with El Niño and La Niña are similar to those of the QBO phases alone (Figs. 9b and 12a, b), a clear poleward wave propagation is seen in deep easterly, lower easterly, and lower westerly QBO phases when combined with neutral ENSO states (Fig. 12c). In addition, variations in vertical wave propagation into the upper stratosphere also play a significant role, strongly contributing to the differences in causal effects. These different responses across ENSO-QBO combinations highlight the importance of considering potential nonlinear interactions between ENSO and QBO when assessing their impacts on the climate system, especially when the sample size is limited.

## Conclusions and discussion

**Fig. 13:**
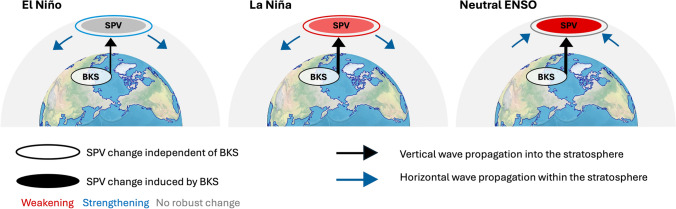
Schematic of ENSO modulation on the BKS-SPV causal linkage. The black vertical arrow depicts upward wave propagation into the stratosphere in response to BKS changes, whereas the blue arrow resembles the wave propagation within the stratosphere. The colour of the outer circle of SPV denotes its ENSO-dependent background state, independent of BKS influence. The colour of the inner circle represents SPV changes in response to changes in BKS. Blue indicates an intensification, red a weakening, and grey for no significant change of the SPV. The SPV response to BKS anomalies is strongest during neutral ENSO conditions, weaker during La Niña, and weakest during El Niño years

In this work, the causal effect of BKS loss on the SPV is investigated in a climate model with a large ensemble (MIROC6). The results indicate that BKS loss causes a weakening of the SPV, as is well known, but the SPV response varies depending on ENSO and QBO phases, reflecting a state-dependent causal effect of BKS on the SPV. In contrast, BKS sensitivity to the background states is minimal. For the causal linkage between autumn mean BKS and winter mean SPV, ENSO states show a robust modulation, where the causal linkage strengthens under La Niña and neutral ENSO states but diminishes under El Niño years (inner circle of SPV in Fig. 13). In addition, the SPV itself shows a strong state dependence on ENSO (outer circle of SPV in Fig. 13), which is independent of BKS. For QBO states, while they have a strong influence on the SPV itself, their modulation of the BKS-SPV causal linkage is minimal, although it is more pronounced when combined with ENSO states. We further see an overall consistent but more robust modulation of ENSO and QBO on the monthly BKS and winter mean SPV linkage, indicating that subseasonal variation of the linkage should be taken into account. Notably, for both ENSO and QBO states, the state-dependence of the BKS-SPV linkage arises both from changes in tropospheric wave forcing and from modulations in wave propagation within the stratosphere (Fig. 13). While it is traditionally understood that ENSO mainly influences the tropospheric wave forcing and QBO predominantly modulates stratospheric wave propagation (e.g., Domeisen et al. 2019; Holton and Tan 1980), the findings from this study suggest that the processes are more complex than this. When combining the influence of ENSO and QBO on the BKS-SPV linkage, the results present a nonlinear modulation instead of a simple additive interaction, suggesting that it is necessary to consider the interactions between ENSO and QBO when assessing their impacts on the climate system. This is in contrast to the effect of ENSO and QBO on the SPV itself (without a BKS anomaly), which in MIROC6 appears to be highly linear (Fig. 5b). Note that previous studies have drawn different conclusions regarding the linearity of the joint influence of ENSO and QBO on the SPV (e.g., Walsh et al. 2022; Wang et al. 2024). These discrepancies likely arise from differences in the climate models and sample size. Therefore, a multi-model large-ensemble analysis will be necessary in future studies to provide a more robust understanding of this linearity.

**Fig. 14:**
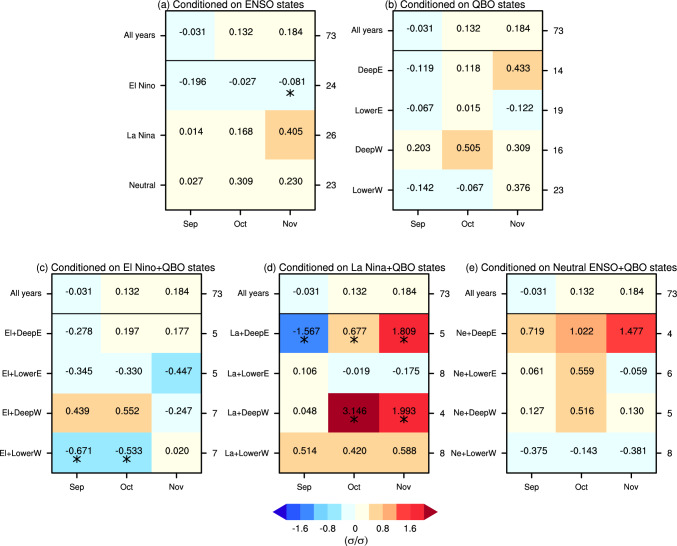
Causal effect of BKS changes on SPV conditioned on different ENSO and QBO states in observations. Same as Figs. 7 and 11, but for the results from the ERA5 reanalysis dataset. Note that the colour bar range is different for visualization purposes compared to Figs. 7 and 11

One question of interest is whether the results from this climate model are consistent with observations. Figure 14 shows the casual effects across different states based on ERA5 reanalysis, with the background states defined in the same way as in the model. While some statistically significant results exist, the very limited sample size introduces considerable uncertainty in the estimated causal effect. This is particularly evident for the ENSO-QBO combined states, where only a few samples are identified (Fig. 14c–e). Therefore, the inclusion or exclusion of even a single sample can lead to substantial changes in the quantitative values. Nevertheless, for ENSO states, El Niño years exhibit a negative causal effect, whereas La Niña years and neutral ENSO years show stronger effects compared to all years. Although, as mentioned earlier, the ENSO-SPV relationship in MIROC6 is opposite to that in ERA5, it does not result in an opposite modulation of the causal effects. This is potentially because the tropospheric circulation response to ENSO is consistent between MIROC6 and ERA5 (Shen et al. 2024), thus, the modulation of the tropospheric wave forcing in response to BKS loss should be similar. Overall, the similarity between the MIROC6 and ERA5 results suggests that it is reasonable to explore this scientific question using MIROC6 even with the opposite ENSO-SPV relationship. On the contrary, the QBO modulation differs more substantially between the observations and the model. While the biases in QBO amplitude and QBO teleconnections might contribute to this difference, this is difficult to assess due to the large uncertainty in the observational estimates. The lack of statistically significant results in the observations further underscores the importance of employing large ensembles to better understand and study climate variability. This is particularly important for understanding apparent model-observation differences (Shaw et al. 2024), including Arctic-midlatitude linkages.

This study combines statistical analysis and physical interpretation to investigate the causal effect of BKS change on SPV. This is achieved by establishing a causal framework grounded in known dynamical linkages and exploring the dynamical mechanisms underlying the statistical relationships. While differing in approach, the results rooted in dynamical mechanisms are overall consistent with previous studies (Labe et al. 2019; Ma et al. 2022; Xu et al. 2024) and provide additional insights on the involved pathways. Given the complexities of climate variability and the distinct limitations of any single approach, it is crucial to utilize various approaches to integrate the efforts across the research community. Here we argue for combining physical and causality-based statistical approaches to identify and quantify teleconnection pathways in the climate system.

## Data Availability

MIROC6 data are available from https://esgf-ui.ceda.ac.uk/cog/projects/esgf-ceda/. HadISST data are available from https://www.metoffice.gov.uk/hadobs/hadisst/data/download.html. ERA5 data are available from https://www.ecmwf.int/en/forecasts/dataset/ecmwf-reanalysis-v5.
